# Electronic epidemiological query on admission: one year results of an e-health based tool designed to risk assessment and infection control

**DOI:** 10.1186/2047-2994-4-S1-P279

**Published:** 2015-06-16

**Authors:** C Palos, A Bispo

**Affiliations:** 1 Infection Control and Antibiotics Committee, Hospital Beatriz Ângelo, Loures, Portugal

## Introduction

Patients admitted to hospitals represent a threat as they may be infected or colonized by Epidemiologically Important Microrganisms (EIM). Their detection allows screening and isolation procedures and should start early, preferably at Emergency Room (ER).

## Objectives

Assessment of the results obtained from 1 year of Electronic Epidemiological Query on Admission (EEQA) fulfillment.

## Methods

EEQA is a 8 question tool obligatory fulfilled at admission. It automatically generates orders (swabs, *Cdiff* toxin and GDH), isolation procedures, e-mails, and ultimately allows detection of colonization/infection by EIM (MRSA, Carbapenem-resistant *Acinetobacter baumanii*, VRE), or infection by *Clostridium difficile* or pulmonary *Mycobacterium tuberculosis*.

## Results

On 2014, 13.893 EEQA were fulfilled. 25% resulted positive. Question (Q)1 (Previous hospital or institutional stay of >3 days, or tracheostomy) contributed to 87,4%. Q2 (EIM present on admission) was positive on 7,5%. Q3 (Dialysis, chemotherapy or immunossupressive therapy on the last 3 months) was detected on 10,8%. Q4 (Pulmonary Tuberculosis suspected or confirmed) contributed to 1,4%. Q5 (Confirmed pneumonia caused by Group A Strepto, Mycoplasma, Adenovirus or H. influenza. Flu, Meningitis… confirmed or suspected) was positive on 0,7% of cases. Q6 (Diarrhea associated with antibiotics administration on the last month, or in the context of contact with other patients with diarrhea, or in patients older than 65y without other cause) represented 3,5%. Q7 (Diarrhea without context defined on question 6, or any exsudative wound or drain without containment) was present on 3,1%. Q8 (Post-transplantaplasia) contributed to 0,2% of positive EEQA.

## Conclusion

EEQA is a simple tool aimed to early detection of high risk patients for infection or colonization by EIM, allowing early and adequate selective screening and isolation procedures according to international guidelines [1], even before micro results are known. This protects healthcare workers, students, other patients and visitors from exposure to these patients, thus minimizing the risk for nosocomial infections.

## Disclosure of interest

None declared.

## References

[B1] Guideline for Isolation Precautions: Preventing Transmission of Infectious Agents in Healthcare Settings 2007 10.1016/j.ajic.2007.10.007 PMC7119119 18068815

